# Calcitonin/Amylin Receptors and Ramps

**DOI:** 10.1100/tsw.2001.442

**Published:** 2001-04-04

**Authors:** Patrick M. Sexton

**Affiliations:** Howard Florey Institute of Experimental Physiology and Medicine, The University of Melbourne, Victoria 3010, Australia

## Abstract

Our understanding of G protein-coupled receptor function has recently expanded to encompass novel protein interactions that underlie both cell surface receptor expression and the exhibited phenotype. The most notable examples are those involving receptor activity modifying proteins (RAMPs). RAMP association with the calcitonin receptor-like receptor (CRLR) traffics this receptor to the cell surface, where individual RAMPs dictate the expression of unique phenotypes.

